# Comparison of Argentinean Saint Louis Encephalitis Virus Non-Epidemic and Epidemic Strain Infections in an Avian Model

**DOI:** 10.1371/journal.pntd.0001177

**Published:** 2011-05-24

**Authors:** Luis Adrián Diaz, Nicole M. Nemeth, Richard A. Bowen, Walter R. Almiron, Marta S. Contigiani

**Affiliations:** 1 Laboratorio Arbovirus, Instituto de Virología “Dr. J. M. Vanella,” Facultad de Ciencias Médicas, Universidad Nacional de Córdoba, Córdoba, Argentina; 2 Colorado State University, Fort Collins, Colorado, United States of America; 3 Centro Investigaciones Entomológicas de Córdoba, Facultad Ciencias Exactas, Físicas y Naturales, Universidad Nacional de Córdoba, Córdoba, Argentina; Centre for Cellular and Molecular Biology (CCMB), India

## Abstract

St. Louis encephalitis virus (SLEV, *Flavivirus*, *Flaviviridae*) is an emerging mosquito-borne pathogen in South America, with human SLEV encephalitis cases reported in Argentina and Brazil. Genotype III strains of SLEV were isolated from *Culex quinquefasciatus* mosquitoes in Cordoba, Argentina in 2005, during the largest SLEV outbreak ever reported in South America. The present study tested the hypothesis that the recent, epidemic SLEV strain exhibits greater virulence in birds as compared with a non-epidemic genotype III strain isolated from mosquitoes in Santa Fe Province 27 years earlier. The observed differences in infection parameters between adult House sparrows (*Passer domesticus*) that were needle-inoculated with either the epidemic or historic SLEV strain were not statistically significant. However, only the House sparrows that were infected with the epidemic strain achieved infectious-level viremia titers sufficient to infect *Cx.* spp. mosquitoes vectors. Furthermore, the vertebrate reservoir competence index values indicated an approximately 3-fold increase in amplification potential of House sparrows infected with the epidemic strain when pre-existing flavivirus-reactive antibodies were present, suggesting the possibility that antibody-dependent enhancement may increase the risk of avian-amplified transmission of SLEV in South America.

## Author Summary

St. Louis encephalitis virus (SLEV, *Flavivirus*, *Flaviviridae*) is an emerging arbovirus in South America, with human SLEV encephalitis cases reported in Argentina and Brazil. Genotype III strains of SLEV were isolated from mosquitoes during the largest SLEV outbreak ever reported in South America (Córdoba, Argentina, 2005). These strains are related to a non-epidemic genotype III SLEV strain isolated in 1979 in Santa Fe Province, Argentina. There is currently no clear explanation for the reemergence of SLEV in Argentina. This study tested the hypothesis that the epidemic strain exhibited greater virulence compared to a non-epidemic genotype III strain in an avian model, the House sparrow (*Passer domesticus*). House sparrows were susceptible to infection with Argentinean SLEV strains; however, the proportion of birds that became detectably viremic was low for both strains. Although no significant difference was detected between both strains, House sparrows inoculated with epidemic strain developed higher and longer viremias than those inoculated with non-epidemic strain. The virus amplification role of House sparrows was apparently enhanced when they had previous flavivirus immunity. The evolutionary/introduction process of a more viremogenic SLEV strain and the immunological interactions among antigenically-related flaviviruses will undoubtedly affect the continued reemergence of SLEV in Argentina.

## Introduction

The geographic distribution of St. Louis encephalitis virus (SLEV, *Flavivirus*, *Flaviviridae*) encompasses tropical, sub-tropical and much of the temperate-tropical zones of the Western Hemisphere and therefore, most of the populated land masses of North and South America [1]. In the United States of America (USA), this virus is known to be naturally maintained by transmission cycles between several *Culex* (*Cx*.) mosquito species and a variety of bird species, including the House sparrow (*Passer domesticus*) [2]. SLEV is an emerging arbovirus in South America, with febrile illness and encephalitis cases reported in Argentina in 2002 and 2005, and in Brazil in 2004 and 2006 [3]–[6].

In Argentina, SLEV reemerged in the central region (i.e., Córdoba and Santa Fe Provinces) in 2002, when two cases of encephalitis and three fever cases were reported in humans (Morales MA and Enria D, unpublished data) [5]. In 2005, 47 laboratory-confirmed clinical cases of SLEV infection, including nine fatalities, were reported from Córdoba Province [7]. Two genotype III SLEV isolates were obtained from *Cx. quinquefasciatus* mosquitoes during this outbreak [4]. Genotype III SLEV was previously isolated from mosquitoes collected in Santa Fe Province 27 years earlier with no human encephalitis cases reported [8]. The cause of the 2005 outbreak remains unknown, but may have derived from virological factors, changes in populations of vectors and/or avian amplifying hosts, and/or environmental conditions [9]–[11]. These were the first reported outbreaks of SLEV-induced encephalitis in South America. By March 2010, the Health Ministry of Buenos Aires Province reported a total of five confirmed and five probable human cases of SLEV [12]. In Brazil, SLEV was identified as the etiologic agent of a small meningoencephalitis outbreak among humans in Sao Paulo State in 2006 [3].

We propose that the recently isolated genotype III strain has pathogenic properties in an avian model, and more specifically, House sparrows. These properties presumably favor epidemic activity relative to the historical genotype III strain that was thought to be enzootic. To test this hypothesis, we evaluated the viremogenic and pathogenic capacities of both recent and historic genotype III isolates from Córdoba in House sparrows. In addition, we evaluated cross-protection conferred by heterologous flavivirus-neutralizing antibodies in a small number of SLEV-challenged House sparrows.

## Methods

### Birds and animal care

House sparrows (hatch-year or older; i.e., adults) were captured in mist nets during January 2007 in Larimer County, Colorado and housed in commercial cages (Safeguard, Inc., New Holland, PA). Mixed bird seed and water were provided *ad libitum*. The maintenance and care of experimental animals in this study complied with institutional guidelines and the National Institutes of Health guidelines for the humane use of laboratory animals. All animal use was conducted at Colorado State University under approval from the Institutional Animal Care and Use Committee (approval 09-137A).

### Virus strains

The CbaAr-4005 (epidemic) and 79V-2533 (non-epidemic) SLEV strains were isolated from pools of adult female *Cx. quinquefasciatus* collected in 2005 in Córdoba Province and *Cx.* (*Culex*) spp. collected in 1978 in Santa Fe Province, Argentina, respectively [4], [8]. The CbaAr-4005 strain had previously been passaged four times and the 79V-2533 strain two times, and all passages were in African Green monkey kidney (Vero) cells.

### Inoculation of birds and sample collection

House sparrows were needle-inoculated subcutaneously over the breast with 3,000 plaque-forming units (PFU) with one of the two SLEV strains (or mock BA-1 inoculation for the negative control group) in 0.1 mL (milliliter) of BA-1 diluent (Hanks M-199 salts, 0.05 M Tris, pH 7.6, 1% bovine serum albumin, 0.35 g/L of sodium bicarbonate, 100 units/mL of penicillin, 100 µg/mL of streptomycin, 1 µg/mL of Fungizone). Seven seronegative birds were inoculated with 79V-2533 (Group A), 8 seronegative (Group B) and 4 flavivirus-seropositive birds (Group C) were inoculated with CbaAr-4005, and 10 birds (Group D) were mock-inoculated with BA-1 to serve as a non-inoculated, morbidity/mortality control group. Following inoculation, birds were monitored for clinical signs (e.g., lethargy, fluffed feathers, decreased activity, and emaciation) every 12 h. From 1–7 days post-inoculation (DPI), 0.1 mL of blood was collected by jugular venipuncture from each bird (including controls) and diluted in 0.45 mL of BA-1 in 2-mL cryovials. The samples were centrifuged for separation of serum (diluted approximately 1∶10) and stored at −80°C until assayed for infectious viral particles.

### Virus assays

The detection and titration of viruses in blood samples was performed using a double-overlay Vero cell plaque assay [13]. The second overlay contained neutral red dye and was added on 5 DPI; plaques were counted on 6 and 7 DPI. Each sample was titrated in duplicate using serial 10-fold dilutions in BA-1 diluent. The detection threshold for SLEV in serum was 10^1.7^ PFU/mL.

### Serology

Pre-inoculation status for flavivirus-reactive antibodies was determined by blocking ELISA (enzyme-linked immunosorbent assay) using the flavivirus group-reactive monoclonal antibody 6B6C-1 and sonicated suspension of CbaAr-4005 antigen [14]. Serum samples collected between 10–14 DPI were assayed using the plaque reduction neutralization test (PRNT) using CbaAr- 4005 on Vero cell monolayer prepared in six-well cell culture plates (Costar Inc, Cambridge, MA) [13]. Between 10 and 14 DPI, all surviving House sparrows were bled (0.6 mL) and whole blood was placed in Microtainer serum separator tubes (Becton Dickinson, Franklin Lakes, NJ), centrifuged for separation of serum, stored at −20°C, and heat inactivated at 56°C for 30 min prior to testing. Serum samples were diluted 1∶10 in BA-1 for antibody screening, and serial 2-fold dilutions in duplicate were used to determine reciprocal endpoint 80% SLEV antibody titers (PRNT_80_). Because West Nile virus (WNV) is endemic in Colorado, flavivirus-reactive antibodies were presumed to be due to previous infection with WNV, which is commonly detected in local House sparrows in northern Colorado (N. Komar, unpublished data).

### Mathematical calculations

Values for vertebrate reservoir competence index (*C*) were calculated according to the formula:
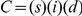
where *s* is susceptibility to infection (a proportion of viremic birds), *i* is the extrapolated mean daily infectiousness (the proportion of feeding *Cx. quinquefasciatus* that are expected to become infected after a viremic blood meal and surviving the extrinsic incubation period; [*i* = 0.5475*log viremia (PFU/ml) – 1.6526], and *d* is mean duration of infectious viremia (in days) [15]. *C* indicates the relative inherent potential for a vertebrate host to amplify a pathogen to sufficient levels to infect vectors. Infectiousness was extrapolated using data published by Mitchell et al. on oral infectivity of *Cx. quinquefasciatus* for 78V-6507 SLEV Argentinean strain. These data indicated an approximate threshold of 10^3.02^ PFU/mL for infectious viremia titers [16].

### Data analyses

Non-SLEV immune sparrows that were inoculated were included in the analyses if they had evidence of infection (i.e., detectable SLEV-viremia between 1–7 DPI and/or seroconverted by 10–14 DPI). Flavivirus-seropositive birds that were inoculated were included in the analyses if they had detectable viremia between 1–7 DPI. To compare susceptibility to infection and mortality among experimental groups, Fisher's exact test was used. Mean durations of viremia and log-transformed mean peak viremia measures were compared using a Poisson generalized linear model and ANOVA, respectively, with significance threshold α = 0.05. Individuals were considered immune if they had detectable anti-SLEV antibodies (PRNT_80_≥10). Individuals were considered refractory (i.e., not susceptible) to infection if they did not show evidence of infection either by detectable viremia titers and/or seroconversion.

## Results

**Figure 1:**
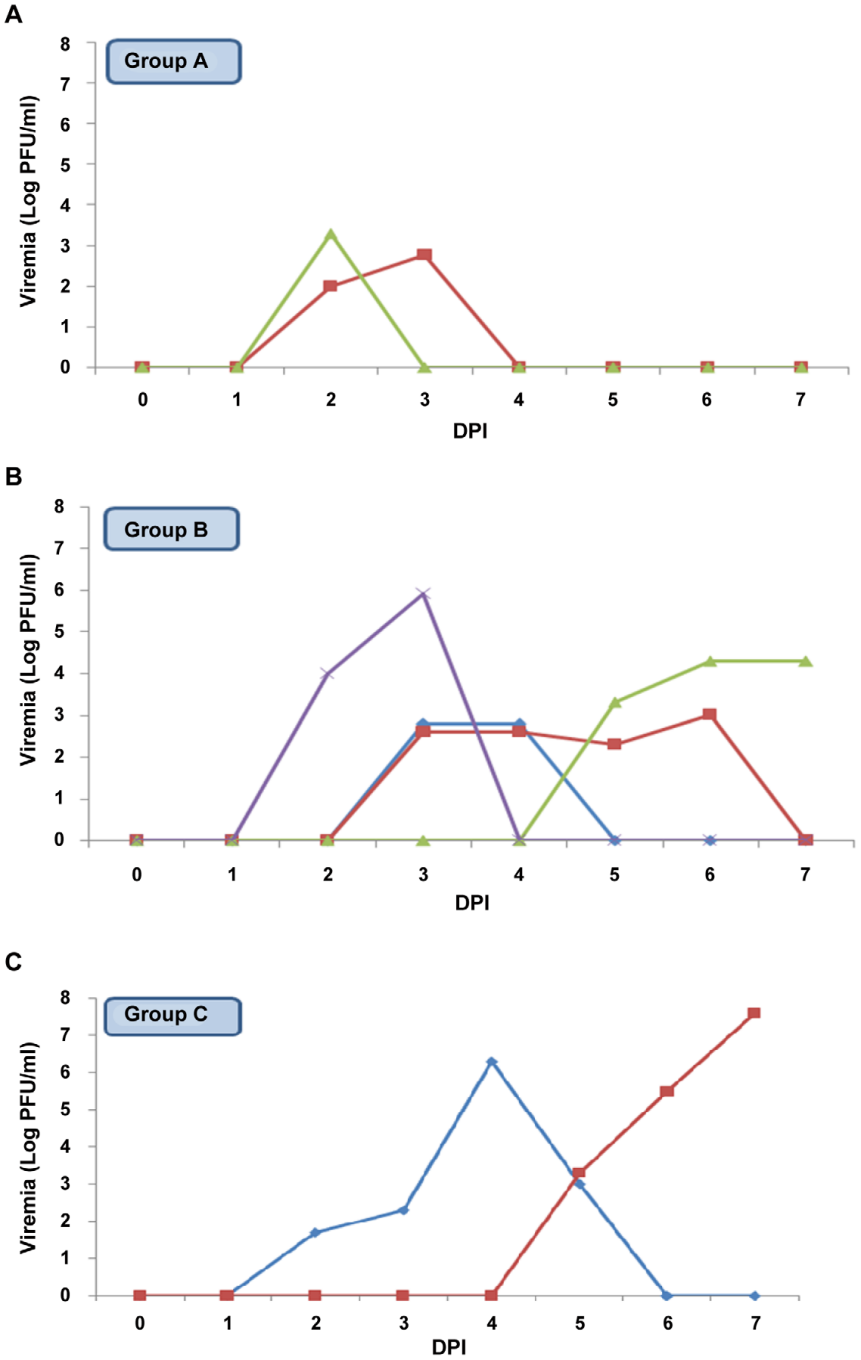
St. Louis encephalitis virus viremia detected in House sparrows inoculated with epidemic and non-epidemic strains. Group A: 79V-2533/naïve, Group B: CbaAr-4005/naïve, Group C: CbaAr-4005/Flavivirus +. DPI: days post-inoculation.

Four of seven House sparrows inoculated with 79V-2533 (Group A) had evidence of infection and were therefore included in the mathematical analyses. Two had detectable viremia (mean peak titer 10^3.1^ PFU/mL serum, mean duration 1.5 days; Table 1) and two were refractory to infection. Six of eight House sparrows inoculated with CbaAr-4005 (Group B) were similarly included in the analyses. Four of these birds had detectable viremia (mean peak titer 10^5.3^ PFU/mL serum, mean duration 2.75 days) and two were refractory. Two of four anti-flavivirus antibody positive birds that had been inoculated with CbaAr-4005 (Group C) were also included in the analyses; these two birds developed detectable viremia (mean peak titer was ≥10^7.1^ PFU/mL serum, mean duration ≥3.5 days (Table 1). The uncertainty in the upper limit of the means for Group C is due to the peak viremia being in one of House sparrows occurring on the last day of sampling (i.e., 7 DPI; Figure 1).

**Table 1:** Viremia and neutralizing antibody titer in House sparrows infected with non-epidemic or epidemic SLEV strains.

Sample #	Group	Viremia^a^				SLEVPRNT_80_ b	Int.
		Min	Max	Mean ± StDev^c^	Duration (days)	14 DPI	
2201	A	-	-	na	0	nd†	UNK
2262	A	-	-	na	0	nd†	UNK
2267	A	2.0	2.8	2.4±0.38	2	nd†	Inf
2269	A	-	-	na	0	N	Ninf
2270	A	-	-	na	0	N	Ninf
2272	A	-	-	na	0	nd†	UNK
2273	A	3.3	3.3	na	1	nd†	Inf
2268	B	3.0	3.0	na	1	nd†	Inf
2275	B	-	-	na	0	N	NInf
2278	B	-	-	na	0	N	NInf
2279	B	-	-	na	0	nd†	UNK
2281	B	2.0	3.0	2.8±0.24	4	20	Inf
2282	B	3.0	4.0	3.7±0.77	3	20	Inf
2283	B	-	-	na	0	nd†	UNK
2287	B	4.0	6.0	5.0±0.11	2	40	Inf
2264	C	-	-	na	0	nd†	UNK
2274	C	1.7	6.3	3.3	4	320	Inf
2276	C	-	-	na	0	nd†	UNK
2288	C	3.3	7.6	5.5	3	nd†	Inf

a Viremia titer expressed as log_10_ of PFU/mL serum. Min = minimum, Max = maximum.

b Plaque reduction neutralization assay against SLEV with an 80% endpoint (PRNT_80_) criterion. DPI = day postinoculation, N = negative, nd† = no data available, individual died before sample collection. Inf = infected, NInf = not infected, UNK = insufficient data. StDev = standard deviation.

c na = not applicable; neither mean nor standard deviation calculated due to lack of data.

Group A: Naïve House sparrows inoculated with 79V-2533 SLEV strain (non-epidemic), Group B: Naïve House sparrows inoculated with CbaAr-4005 SLEV strain (epidemic) and Group C: Anti-flavivirus antibody positive House sparrows inoculated with epidemic SLEV strain.

Mortality was observed in all four of the study groups, including the non-inoculated control group. Through 7 DPI, mortality occurred in 3 of 7 (Group A), 4 of 8 (Group B), 2 of 4 (Group C), and 3 of 10 (Control Group) birds in the four groups. The rates did not differ significantly among any of the groups (*P*>0.5).

Infection parameters (i.e., susceptibility to infection, mean duration of viremia, and mean peak viremia titers) were compared between House sparrows in Groups A and B, A and C, and B and C. Sample sizes were insufficient to detect statistically significant differences in any of these pair-wise comparisons. However, considering that a threshold viremia titer for infection of vector mosquitoes with the 79V-2533 SLEV strain could be estimated from published data, and that the viremia titers observed in the experimental groups appeared to be skewed in relation to this threshold, we used these data to assess the biological significance of the data. A rough estimate of the relative reservoir competence of House sparrows in each experimental group indicated that adult House sparrows were not competent amplifying hosts for the non-epidemic SLEV strain (Group A). On the other hand, House sparrows inoculated with the epidemic SLEV strain (Group B) were theoretically able to produce 10 times more infectious mosquitoes than those inoculated with the non-epidemic strain (Table 2). House sparrows with prior flavivirus infection (Group C) would infect three mosquitoes for every one mosquito infected by flavivirus-naïve House sparrows (Group B) inoculated with the epidemic SLEV strain (CbaAr-4005) (Table 2).

**Table 2:** Potential transmission by House sparrows for epidemic and non-epidemic St. Louis encephalitis strains from Argentina.

Viral Strain/Treatment	Susceptibility(*s*)^a^	Infectiousness(*i*)	Duration(*d*)^b^	Comp. index (*ci*)^c^	R. comp. index
79V-2533/naïve (A)	0.5	0.15	1.5	0.11	1
CbaAr-4005/naïve (B)	0.7	0.58	2.8	1.14	10^AvsB^
CbaAr-4005/Flavi+ (C)^d^	1	1	3.5	3.5	32^AvsC^3^BvsC^

a *s* = mean ratio of viremic individuals/inoculated individuals.

b *d* = mean duration of viremia in days.

c *Ci* (host competence index) = *s* * *i* * *d*. *i* = infectiousness for a given bird species.

d House sparrows with previous anti-flavivirus antibodies.

## Discussion

Variation in biological characteristics (i.e., viremia profiles, neuroinvasiveness, virulence, and pathogenicity) among SLEV strains has been previously described [17], [18], [19]. Bowen *et al.* [18] compared infection parameters for 44 strains of SLEV, including 14 from South America, in adult and juvenile House sparrows, and found that strains could be categorized as low, intermediate, or high viremogenic capacity [18]. In the present study, these same infection parameters in adult House sparrows were compared for a non-epidemic strain (presumably not pathogenic in humans) from Argentina and a novel, epidemic strain isolated during the first SLEV outbreak ever recognized in South America. The two strains were isolated from the same subtropical region of northern Argentina, but 27 years apart. The recently discovered strain (CbaAr-4005) is associated with an outbreak of human encephalitis resulting in nine fatalities in Córdoba, Argentina. Despite alignment of the genomes of these two strains within the same genotype, the epidemic strain appears to be more viremogenic than the non-epidemic strain in our model avian host. Comparison of the complete genome sequences of the two strains revealed an amino acid difference at position 249 in the NS3 protein. This is the same position as described for bird-virulent strains of WNV (T249P) [20].

Low or undetectable viremia titers among House sparrows in the present study may have lessened the likelihood of detection of statistically significant differences. The sample sizes were similar to those used by Bowen *et al.* [18], yet applying their criteria for categories of viremogenic capacity failed to differentiate the two Argentinean strains into either the high or low viremogenic categories. However, using previously published data [16] for vector competence of *Cx. quinquefasciatus* mosquitoes, data obtained in the present study indicated that adult House sparrows infected with the non-epidemic strain were incompetent amplifiers of SLEV. House sparrows inoculated with the epidemic strain were theoretically competent, based on a biologically significant difference in the observed data for the two strains.

Viremia profiles varied among individuals, including among those inoculated with the same SLEV strain. Intrinsic avian factors such as genetics, age, and immunocompetence are likely to variably-affect host responses to infection. The inverse effect of age over resulting viremia profiles after arbovirus infection (i.e., older individuals develop lower viremia titers than younger individuals) has been well documented [18], [21], [22], [23]. Although all House sparrows in the present study were considered adults, we could not further specify age beyond >1 year of age, so that actual ages may have varied widely and therefore influenced viremic responses. In addition, while we observed no visible health effects in these birds during the pre-inoculation period, underlying health conditions may also have affected their responses to infection. In addition, previous studies have identified a specific gene (i.e., the Oas1b gene; 2′-5′ oligoadenylate synthetases) as a determining factor for resistance to infection in animals (i.e., humans, mice, chickens and horses) [24], [25], [26]. Genetic variability in this gene and possibly others among inoculated House sparrows in this study could have also caused differences in responses to infection.

Mortality was observed in each of the four study groups, one of which was the non-inoculated control group. No statistical difference was detected in mortality among groups. Therefore, we believe that the observed mortality was attributable to captivity, handling stress, and possibly the aforementioned factors, and not attributable to SLEV infection. Further, SLEV is not historically believed to cause morbidity and mortality in birds either experimentally or naturally infected [2], [22], [27], [28].

The relatively small proportion of House sparrows with detectable viremia titers, which were generally low, could indicate co-adaptation of South American SLEV strains among the resident avifauna. House sparrows have a broad geographic range and are considered the main amplifying host of SLEV in south and central USA, and House finches (*Carpodacus mexicanus*) the main host for SLEV strains in the western coast of the USA (e.g., California) [2]. Argentinean SLEV strains are not well amplified by resident House sparrows in Argentina. [LA Diaz, unpublished data] However, Picui ground doves (*Columbina picui*) and Eared doves (*Zenaida auriculata*) are amplifying hosts for SLEV strains in Argentina [Diaz LA, unpublished data], further supporting the idea that SLEV strains have become adapted to their respective resident bird populations in both the USA and Argentina.

In the present study, House sparrows with evidence of previous (i.e., natural) flavivirus infection that were subsequently inoculated with SLEV strain CbaAr-4005 developed higher viremia titers of longer duration than naïve House sparrows, as evidenced by a 3-fold greater reservoir competence in the former. While this assessment is based on a small number of available sparrows with previous flavivirus immunity and more data are needed, it suggests that SLEV activity may be enhanced by previous circulation of WNV or other flaviviruses among avian host populations. Pre-existing flavivirus-reactive antibodies in House sparrows (perhaps homologous anti-SLEV or heterologous anti-WNV) may potentiate subsequent SLEV infection, as indicated by the higher resulting viremia titers in these birds following challenge. Ludwig et al. reported a similar finding in a laboratory controlled experimental SLEV infection of House sparrow chicks circulating various levels of homologous maternal antibodies [29]. Viremia titers of greater magnitude and duration were observed in nestling House sparrows from SLEV-inoculated mothers versus nestlings from naïve mothers. As maternal SLEV-neutralizing antibody titers wane in nestling birds, antibody-mediated amplification of serum virus titers may result following SLEV infection. Antibody-dependent enhancement of *Flavivirus* infections is a well-known phenomenon observed among humans with secondary dengue virus infection [30]. However, House finches (*Carpodacus mexicanus*) with pre-existing antibodies to WNV were protected from experimental challenge with a North American strain of SLEV [31].

With many closely-related flaviviruses circulating in South America, the possibility of antibody-dependent enhancement of SLEV infections in birds requires further investigation, especially if an association with emergence of SLEV epidemic activity is to be corroborated. In Brazil, for example, there are at least ten circulating flaviviruses, including Bussuquara, Cacipacoré, Dengue (serotypes 1 to 4), Igaupe, Ilheus, Rocío, SLE, and yellow fever viruses [32]. In addition, WNV and SLEV are now sympatric throughout the Americas from southern Canada to central Argentina, and co-circulation has been observed in some locations with active SLEV surveillance programs such as Florida, Texas and California [33], [34]. WNV has been active in Argentina since at least 2004, as indicated by detection of specific WNV-reactive neutralizing antibodies in sera collected from a Rufous hornero (*Furnarius rufus*), a resident passerine, sampled on 5 January 2005 [35]. The appearance of WNV in central Argentina shortly before an unprecedented encephalitis epidemic caused by SLEV may not have been a coincidence, but rather potentially the consequence of the same antibody-dependent enhancement that we observed in our small study.

Currently, there is no definitive explanation for the reemergence of SLEV in Argentina. Two possible explanations gain some support from data in the present study. The first is the evolution or introduction of a SLEV strain with increased viremogenicity. The higher viremia titers generated by infections with the CbaAr-4005 strain in local amplifier species such as the Eared dove [36] will theoretically lead to increased numbers of infected vectors. Furthermore, this epidemic strain appears to broaden the number of avian species that are likely to be competent amplifying hosts relative to the non-epidemic 79V-2533 strain. The identification of a limited number of specific amino acid substitutions between the two genotype III strains used in the present study helps direct future research to identify molecular virulence factors [37]. The second possible explanation is that recent introduction of WNV into the region has boosted the reservoir competence of local avian reservoir hosts for SLEV through antibody-mediated enhancement. WNV activity in the USA had a sobering impact on wild bird populations, resulting in the deaths of millions of birds, while SLEV does not cause avian mortality. However, since the introduction of WNV to Argentina in 2004, there have been no reports of associated avian mortalities. These explanations are not mutually exclusive, and other factors may be involved. In order to provide more support for our findings, further studies should focus on the immunological interactions among antigenically-related flaviviruses in birds and other potential amplifying hosts.

The possibility that this newly discovered epidemic SLEV strain may spread within Argentina as well as to other regions of Central and South America represents an important public health threat. In early 2010, SLEV-associated encephalitis cases in humans were reported in Buenos Aires Province [12]. During this outbreak, molecular detections of SLEV were made. BLAST analyses revealed that the nucleotide sequence of the 232 pb NS5 polymerase amplified fragment had 100% homology with that of the epidemic CbaAr-4005 SLEV strain (GenBank accession # FJ753286.1) (L. Valinotto, unpublished data), indicating an epidemic association with a genotype III SLEV strain. Surveillance for vector-borne pathogens remains an unattended civic priority across the globe. In the absence of early detection through environmental surveillance, clinicians should be on alert for neurologic syndromes in human patients attributed to this novel strain of SLEV.

## References

[1] Monath TP, Monath TP (1980). Epidemiology.. St. Louis encephalitis.

[2] Reisen W (2003). Epidemiology of St. Louis encephalitis virus.. Adv Virus Res.

[3] Mondini A, Soares Cardeal IL, Lázaro E, Nunes SH (2007). Saint Louis encephalitis virus, Brazil.. Emerg Infect Dis.

[4] Díaz LA, Ré V, Almirón WR, Farías A, Vázquez A (2006). Genotype III Saint Louis encephalitis virus outbreak, Argentina, 2005.. Emerg Infect Dis.

[5] Spinsanti L, Basquiera AL, Bulacio S, Somale V, Kim SC (2003). St. Louis Encephalitis in Argentina: the First Case Reported in the Last Seventeen Years.. Emerg Infect Dis.

[6] Rocco IM, Santos CL, Bisordi I, Petrella SM, Pereira LE (2005). St. Louis encephalitis virus: first isolation from a human in São Paulo State, Brazil.. Rev Inst Med Trop Sao Paulo.

[7] Spinsanti LI, Díaz LA, Glatstein N, Arselán S, Morales MA (2008). Human outbreak of St. Louis encephalitis detected in Argentina, 2005.. J Clin Virol.

[8] Mitchell CJ, Monath TP, Sabattini MS, Cropp CB, Daffner JF (1985). Arbovirus investigations in Argentina, 1977–1980. II. Arthropod collections and virus isolations from argentine mosquitoes.. Am J Trop Med Hyg.

[9] Kramer LD, Presser SB, Hardy JL, Jackson AO (1997). Genotypic and phenotypic variation of selected Saint Louis encephalitis viral strains isolated in California.. Am J Trop Med Hyg.

[10] Solomon T, Mallewa M (2001). Dengue and other emerging flaviviruses.. J Infect.

[11] Gubler DJ (2002). The global emergence/resurgence of arboviral disease as public health problems.. Arch Med Res.

[12] PAHO website. Organización Panamericana de la Salud (2010). Alerta epidemiológica: nuevos casos confirmados de encefalitis de San Luis en ciudad y provincia de Buenos Aires, República Argentina. Riesgo de diseminación.. http://new.paho.org/hq/index.php?option=com_docman&task=doc_download&gid=5187&Itemid=.

[13] Beaty BJ, Calisher CH, Shope RE, Lennette EH, Lennette DA, Lennette ET (1995). Diagnostic procedures for viral, rickettsial, and chlamydial infections.. Arboviruses.

[14] Blitvich BJ, Marlenee NL, Hall RA, Calisher CH, Bowen RA (2003). Epitope-blocking enzyme-linked immunosorbent assays for the detection of serum antibodies to West Nile virus in multiple avian species.. J Clin Microbiol.

[15] Komar N, Dohm DJ, Turell MJ, Spielman A (1999). Eastern equine encephalitis virus in birds: relative competence of European starlings (Sturnus vulgaris).. Am J Trop Med Hyg.

[16] Mitchell CJ, Monath TP, Sabattini MS (1980). Transmission of St. Louis encephalitis virus from Argentina by mosquitoes of the Culex pipiens (Diptera: Culicidae) complex.. J Med Entomol.

[17] Mitchell CJ, Gubler DJ, Monath TP (1983). Variation in infectivity of Saint Louis Encephalitis viral strains for Culex pipiens quinquefasciatus (Diptera: Culicidae).. J Med Entomol.

[18] Bowen GS, Monath TP, Kemp GE, Kerschner JH, Kirk LJ (1980). Geographic variation among St. Louis encephalitis virus strains in the viremic responses of avian hosts.. Am J Trop Med Hyg.

[19] Monath TP, Cropp CB, Bowen GS, Kemp GE, Mitchel CJ (1980). Variation in virulence for mice and rhesus monkeys among St. Louis encephalitis virus strains of different origin.. Am J Trop Med Hyg.

[20] Brault AC, Huang CY, Langevin SA, Kinney RM, Bowen RA (2007). A single positively selected West Nile viral mutation confers increased virogenesis in American crows.. Nat Genet.

[21] Trent DW, Grant JA, Vorndam AV, Cropp BC, Kemp GE (1980). Variaton among strains of St. Louis encephalitis virus: basis for a genetic, pathogenetic and epidemiological classification.. Annals NY Acad Sci.

[22] Reisen WK, Chiles RE, Martinez VM, Fang Y, Green EN (2004). Encephalitis virus persistence in California birds: experimental infections in mourning doves (Zenaidura macroura).. J Med Entomol.

[23] Mahmood F, Chiles RE, Fang Y, Barker CM, Reisen WK (2004). Role of nestling mourning doves and house finches as amplifying hosts of St. Louis encephalitis virus.. J Med Entomol.

[24] Tatsumi R, Kazushige H, Sadanori S, Masaki W, Takao N (2000). 2–5 oligoadenylate synthetase gene in chicken: gene structure, distribution of alleles and their expression.. Biochem Bioph Acta.

[25] Brinton MA, Perelygin AA (2003). Genetic resistance to flaviviruses.. Adv Virus Res.

[26] Rios JJ, Fleming JG, Bryant UK, Carter CN, Huber JC (2010). OAS1 polymorphisms are associated with susceptibility to West Nile encephalitis in horses.. PLoS One.

[27] McLean RG, Bowen GS, Monath TP (1980). Vertebrate hosts.. “St. Louis encephalitis”.

[28] Reisen WK, Chiles RE, Martinez VM, Fang Y, Green EN (2003). Experimental infection of California bids with western equine encephalomyelitis and St. Louis encephalitis viruses.. J Med Entomol.

[29] Ludwig GV, Cook RS, McLean RG, Francy DB (1986). Viremic enhancement due to transovarially acquired antibodies to St. Louis encephalitis virus in birds.. J Wild Dis.

[30] Halstead SB (2003). Neutralization and antibody-dependent enhancement of dengue viruses.. Adv Virus Res.

[31] Fang Y, Reisen WK (2006). Previous infection with West Nile or St. Louis encephalitis viruses provides cross protection during reinfection in house finches.. Am J Trop Med Hyg.

[32] Figueiredo LT (2000). The Brazilian flaviviruses.. Microbes and Infection.

[33] Reisen WK, Lothrop HD, Wheeler SS, Kennsington M, Gutierrez A (2008). Persistent West Nile virus transmission and the apparent displacement St. Louis encephalitis virus in southeastern Califronia, 2003–2006.. J Med Entomol.

[34] Brault AC (2009). Changing patterns of West Nile virus transmission: altered vector competence and host susceptibility.. Vet Res.

[35] Diaz LA, Komar N, Visintin A, Dantur Juri MJ, Stein M (2008). West Nile virus in birds, Argentina.. Emerg Infect Dis.

[36] Diaz LA, Occelli M, Almeida FL, Almirón WR, Contigiani MS (2008). Eared dove (Zenaida auriculata, Columbidae) as host for St. Louis encephalitis virus (Flaviviridae, Flavivirus).. Vector Borne Zoonotic Dis.

[37] Diaz LA, Goñi S, Iserte J, Logue C, Singh A (2010). Molecular characterization of epidemic and non-epidemic St. Louis encephalitis virus (SLEV) strains isolated in Argentina.. Am J Trop Med Hyg.

